# Hoxd13/Bmp2-mediated mechanism involved in zebrafish finfold design

**DOI:** 10.1038/s41598-021-86621-4

**Published:** 2021-03-30

**Authors:** João Castro, Vanessa Beviano, Ana Paço, Joana Leitão-Castro, Francisco Cadete, Miguel Francisco, Renata Freitas

**Affiliations:** 1 I3S, Institute for Innovation and Health Research, University of Porto, Porto, Portugal; 2 Cell Growth and Differentiation Group, IBMC/I3S, Rua Alfredo Allen 208, 4200-135 Porto, Portugal

**Keywords:** Evolutionary developmental biology, Morphogenesis, Developmental biology, Evolution

## Abstract

The overexpression of *hoxd13a* during zebrafish fin development causes distal endochondral expansion and simultaneous reduction of the finfold, mimicking the major events thought to have happened during the fin-to-limb transition in Vertebrates. We investigated the effect of *hoxd13a *overexpression on putative downstream targets and found it to cause downregulation of proximal fin identity markers (*meis1* and *emx2*) and upregulation of genes involved in skeletogenesis/patterning (*fbn1*, *dacha*) and AER/Finfold maintenance (*bmps*). We then show that *bmp2b* overexpression leads to finfold reduction, recapitulating the phenotype observed in *hoxd13a*-overexpressing fins. In addition, we show that during the development of the long finfold in leo^t1^/lof^dt1^ mutants, *hoxd13a* and *bmp2b* are downregulated. Our results suggest that modulation of the transcription factor Hoxd13 during evolution may have been involved in finfold reduction through regulation of the Bmp signalling that then activated apoptotic mechanisms impairing finfold elongation.

## Introduction

The evolutionary transition from fish fins to tetrapod limbs involved sequential expansion and elaboration of the endoskeleton and simultaneous reduction of the distal ectodermal finfold (FF), formed by elongation of the embryonic apical ectodermal ridge (AER)^1–3^. This embryonic structure has signaling activity involved in maintaining cell proliferation in the underlying mesenchyme and contributing to the outgrowth and patterning of fins and limbs^4–6^. However, striking differences were detected in the developmental mechanisms of the AER in fish fins and tetrapod limbs^7–9^. During fin development, the AER is rapidly converted into a FF^10,11^, which is then colonized by mesenchymal cells that differentiate into actinotrichia and maturate into lepidotrichia (bony fin-rays)^10,12,13^. However, during limb development, the AER fails to elongate and its signaling activity persists up to the differentiation of the autopod^4,9,14^. The distinct behavior of the AER in fish fins and tetrapods limbs led Thorogood^15^ to propose the “clock model” to explain the fin-to-limb transition, according to which early FF formation hinders mesenchyme expansion, which is required to further promote distal endoskeletal formation^15^. Thus, a heterochronic shift in the AER-to-FF transition was proposed as essential for the origin of limbs, with the transition occurring earlier in actinopterygians, later in sarcopterygians, and being absent in tetrapods^7,15,16^. However, the genetic drivers of this heterochronic shift remain largely unknown.

Genes in the 5′ end of the HoxD cluster, such as *Hoxd13*, were shown to be essential for autopod formation in tetrapods^17–19^, in which they are expressed in two distinct phases activated either by *cis*-regulatory regions located in the telomeric or centromeric flanking regions of the HoxD cluster^18,20,21^. In the first phase, 5′Hoxd genes are transcribed exclusively in the posterior mesenchyme of the limb buds, while in the second phase, their expression domain expands distally and anteriorly occupying the entire prospective autopod region^22,23^.

Chondrichthyans^24^, basal actinopterygians^25^, teleosts^26^, and lungfishes^27^ were shown to have also two phases of 5′HoxD gene expression during fin development, however, their expression patterns never fully recapitulate the ones observed during tetrapod limb development^28,29^. These observations lead to the hypothesis that transcriptional modulation of 5′HoxD genes (and *Hoxd13* in particular), through the addition of enhancer modules, was crucial for the formation of novel distal endoskeletal elements during limb evolution^18,30–32^. Freitas and colleagues address this idea using zebrafish as a pre-tetrapod representative, which lacks particular enhancer units in the telomeric landscape of the HoxD cluster highly conserved in tetrapods^32^. Mimicking the function of these enhancer units, they induced *hoxd13a* over-expression during fin development and observed expansion of the chondrogenic tissue distally and concomitant reduction of the finfold, a phenotype that resembles the morphological transformations thought to have happened during the fin-to-limb transition^28,32^. Likewise, *Actinodin1/2* knockdown during zebrafish development fin (*and1*, *and2*) interferes with finfold formation and leads to the expression of genes involved in tetrapod digit development^13^. However, *hoxd13a* homozygous null embryos exhibit normal fins, with no visible shortening of the fin rays in adulthood, while double *hoxa13a/hoxa13b* or triple *hoxa13a/hoxa13b/hoxd13a* knockouts lack finfold development, possible due to a deficit in migration of mesenchymal cells to the finfold impairing dermal skeleton development^33^. Taken together, these data suggest that early silencing of *hoxd13a* does not affect finfold development, however time-specific overexpression, 30–32 h post-fertilization (hpf), is able to induce finfold shortening. Nevertheless, tantalizing questions remain unresolved, such as which *Hoxd13*-associated mechanisms are responsible for this phenotype.

Here we used a zebrafish line, allowing time-specific overexpression of *hoxd13a*, to evaluate the impact on putative downstream targets^32,34^. One of these putative targets, *bmp2b*, shown to have expression levels affected by *hoxd13a* overexpression, was then further investigated. To this end, we generated a transgenic zebrafish line that allows *bmp2b* overexpression specifically at 32hpf, as performed for *hoxd13a*, which caused a significant reduction of the finfold, accompanied by an increase in cell death. To further explore the idea that *bmp2b* may influence finfold size, we undertook gene expression analyses for *bmp2b* and *hoxd13a* in zebrafish mutants leo^t1^/lof^dt1^, characterized by long finfolds^35^. These animals carry the leo^t1^ recessive mutation located in *connexin41.8* (*cx41.8*)^36^ and the lof^dt2^ dominant mutation located in *Kcc4a,* encoding K + -Cl − co-transporter and causing long finfolds^37^. We found that during the development of their long finfolds, both *bmp2b* and *hoxd13a* are less expressed than in Wild-Type fins (WT).

We propose an evolutionary model in which increased levels of *Hoxd13* expression during fin development may have caused higher expression levels of *Bmps* at the distal margin of fins, promoting apoptosis and contradicting finfold elongation. In addition, higher levels of *Hoxd13* may have also promoted a skeletogenic fate in the most distal cells by causing ectopic expression of *fbn1* and *dach* genes.

## Results

### *hoxd13a* overexpression during fin development affects the expression of putative targets

Freitas and colleagues performed three independent assays aiming to overexpress *hoxd13*a in a spatially or temporally controlled manner^32^. Overexpression of *hoxd13a* at 32hpf, when its expression is known to expand anteriorly in zebrafish fins^26^, was achieved by generating transgenics in which *hoxd13a* was placed under the control of a hormone-inducible promoter, a *col2a1* promoter, or a heat-shock protein 70 promoter (hsp70). Transgenic fish carrying any of those 3 constructs display the same morphological and gene expression phenotype, characterized by distal expression expansion of chondrogenic markers and a simultaneous decrease in the expression of finfold markers, resulting in a distal expansion of the endochondral plate and reduction of the finfold in transgenic fish.

We started by determining if the decedents of the original transgenic line, which was outcrossed with AB WT animals, still display the same fin phenotypes reported by Freitas and colleagues after heat-shock induction^32^. Adjusting the exposure time of the heat-shock treatments, we obtained embryos that displayed finfold reduction or truncation associated with the downregulation of the finfold marker *and1,* detected by in situ hybridization (ISH, n = 10) (Supplementary Information: Fig. 1A). In addition, we also dissected fins (n = 100) from wild type (Wt) and transgenic embryos (*hsp70:hoxd13a*) to undertake a quantitative gene expression evaluation throughout development, regarding *hoxd13a, and1,* and *fgf8*, an additional finfold marker (Supplementary Information: Fig. 1B,C). We detected much higher levels of *hoxd13a* transcripts in the transgenic fins than in the Wt controls at three distinct time points: 56hpf, 85hpf, and 115hpf. At the same stages, the expression of *and1* and *fgf8* was significantly lower in the transgenic fins than in the controls, which was in agreement with the identified phenotypes.

**Figure 1:**
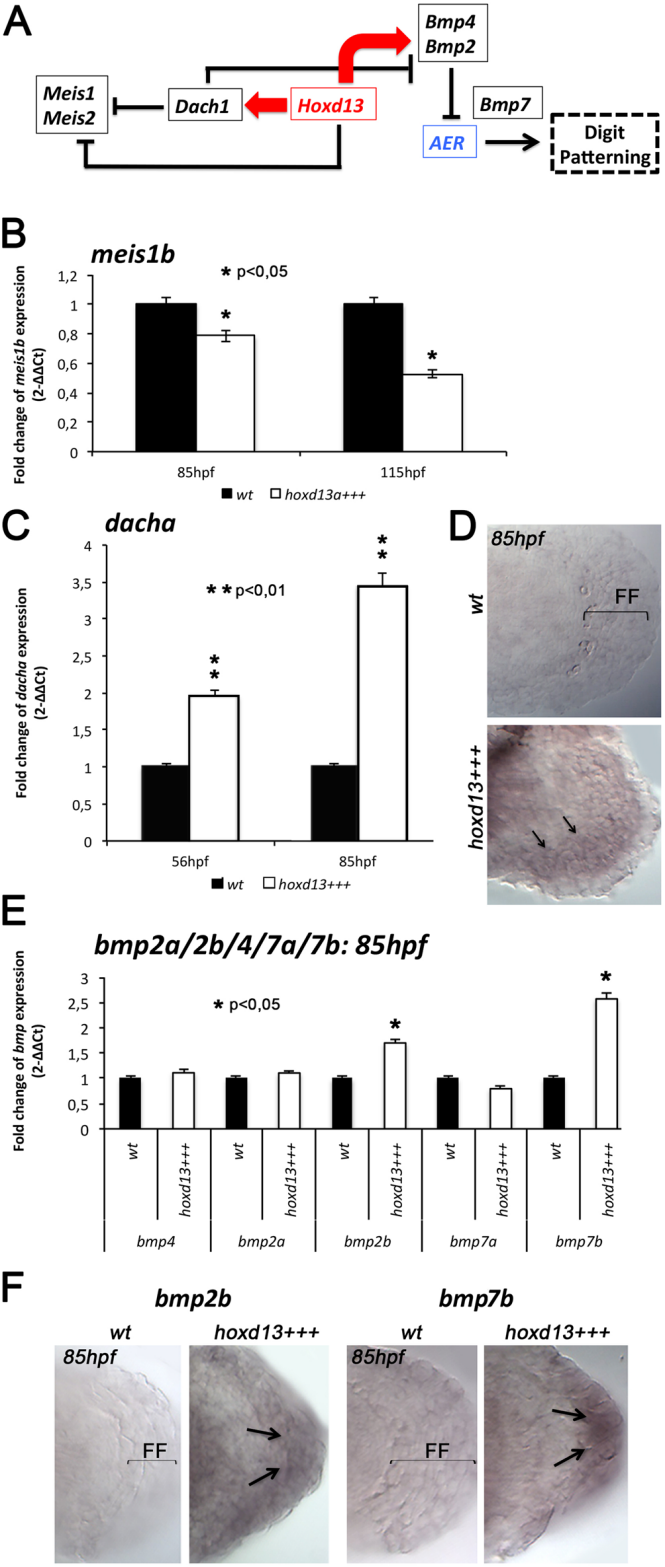
Expression levels of *hoxd13a* putative targets in *hoxd13a*-overexpressing fins (*hoxd13*+++) and controls (*Wt*) analyzed by RT-qPCR **(B,C,E)** and ISH **(D,F,G). (A)** Central role of the transcription factor Hoxd13 in the distal patterning of tetrapod limbs, based in Salsi and colleagues^6^. (**B)** *meis1b* appears downregulation in *hoxd13*+++ fins in comparison with *Wt* at 85 and 115 hpf. (**C,D)** *dachA* presents higher expression in *hoxd13*+++ fins than in *Wt* controls at 56 and 85 hpf **(C)** and is ectopically expressed in the distal margin of the transgenic fins at 85hpf (arrows), under the remaining finfold (FF) **(D)**. (**E,F)** Expression analyses of *bmp2a*, *bmp2b*, *bmp4*, *bmp7a,* and *bmp7b* reveal upregulation of *bmp2b* and *bmp7b* in *hoxd13*+++ fins **(E)**, which are ectopically expressed in the remaining finfold. Statistical significance evaluated by unpaired t-test: *p < 0.05; **p < 0.01.

Using dissected *hoxd13a*-overexpressing fins and controls, we then evaluated the expression of a set of putative downstream targets, identified in a Chip-to-chip assay performed in mice embryonic limbs (*Meis1*, *Dach1, Bmp2, Bmp4, Barx1, Fbn1,* and *Emx2*)^34^ (Fig. 1A). We found that *meis1b is* significantly downregulated in *hsp70:hoxd13a* fins at 85hpf and 115hpf (Fig. 1B), which suggests that *hoxd13a* overexpression may have caused *meis1b* inhibition*.* The tetrapod counterpart, *Meis1,* is required to establish proximal identity during limb patterning leading to the differentiation of the stylopod^38,39^ and found to be downregulated by Hoxd13 in chick limb buds^38^. In addition, the analyses of the three *Dach1* zebrafish orthologous revealed upregulation of *dacha* as early as 56hpf in the transgenic fins (Fig. 1C), and its expression is maintained in the most distal mesenchyme in *hoxd13a*-overexpressing fins at 85hpf, contrarily to what is observed in WT controls (Fig. 1D). Thus, *hoxd13a* overexpression appears to positively impact *dacha* expression*,* which according to the identified role of its orthologous in tetrapods may lead to *meis1b* repression^34^. In addition, *dacha* may also work as a Bmp antagonist regulating finfold formation, as suggested to happen during chick limb development in which Dach1 is able to repress the BMP-mediated transcriptional control regulating the formation of the AER^40^.

**Figure 2:**
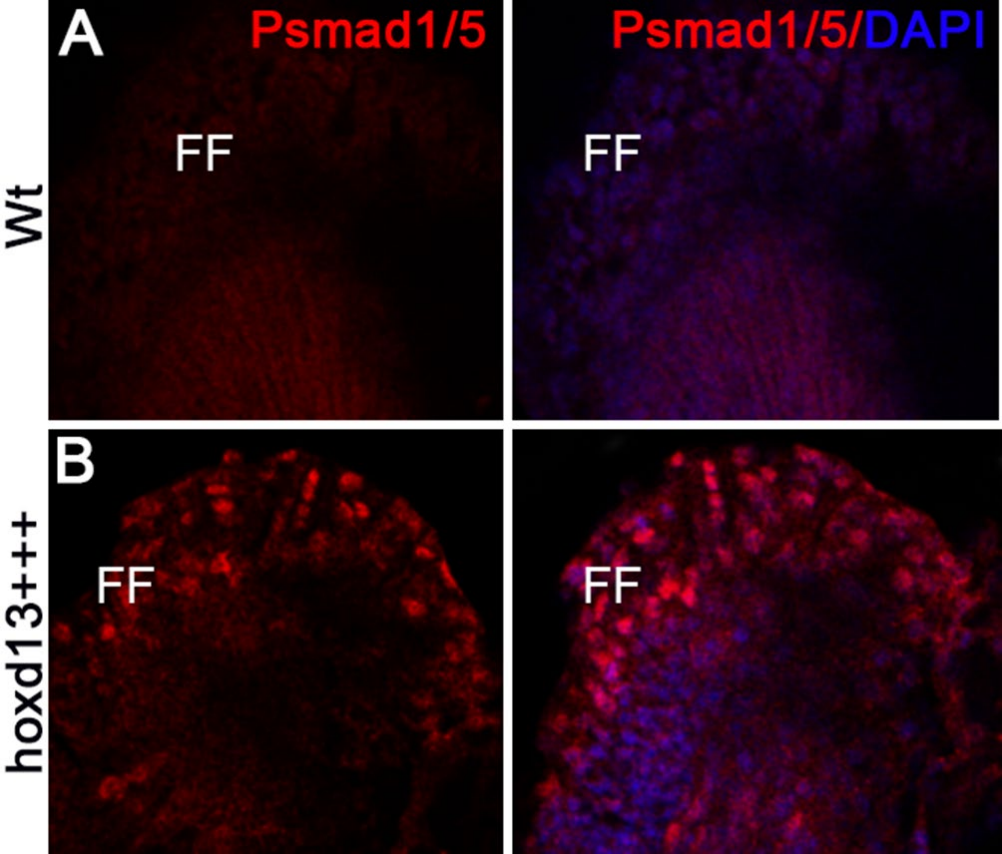
Anti-phospho-smad1/5 immunostaining (Psmad1/5) at 86hpf in wild-type **(A) ***hoxd13a*-overexpressing fins** (B).** Note intense positive staining in the remaining finfold (FF) of the *hoxd13a*-overexpressing fins (*hoxd13*+++), which is not detectable in the wild-type condition (*Wt*).

In order to evaluate the impact of *hoxd13a* overexpression in the BMP-signaling, which was implicated in the abnormal expansion of the AER in chick embryos^41^, we analyzed the expression of *Bmp2*, *Bmp4,* and *Bmp7* orthologous during fin development (Fig. 1E,F). We found significant upregulation of *bmp2b* and *bmp7b* at 85hpf, which appears ectopically expressed in the distal border of the transgenic fins (Fig. 1F). Further evidence for upregulation of the Bmp signaling in *hoxd13a*-overexpressing fins emerged from the immunostaining with anti-Psmad1/5 antibody, which revealed Psamd1/5 positive cells in the modified finfold from the transgenic fins not observed in the wild-type condition (Fig. 2).

We also evaluated the expression of *fbn1*, a gene involved in skeletogenesis in tetrapods that encodes a major component of the extracellular microfibrils^42^. Our analyses revealed that *fbn1* is expressed in higher levels in *hoxd13a*-overexpressing fins than in controls at 56hpf (Supplementary Information: Fig. 2A). This suggests that *hoxd13a* overexpression may interfere with the formation of extracellular microfibrils at the margin of zebrafish fins. In addition, we observed lower expression levels of *emx2* and *barx1* in *hoxd13a*-overexpressing fins at three distinct developmental stages (Supplementary Information: Fig. 2B)*,* genes involved in proximal fin patterning^43^. These data suggest that *hoxd13* overexpression may inhibit proximal identity signals during fin development, as proposed by Freitas and colleagues^32^.

### Overexpression of *bmp2b* during fin development causes finfold reduction

**Figure 3:**
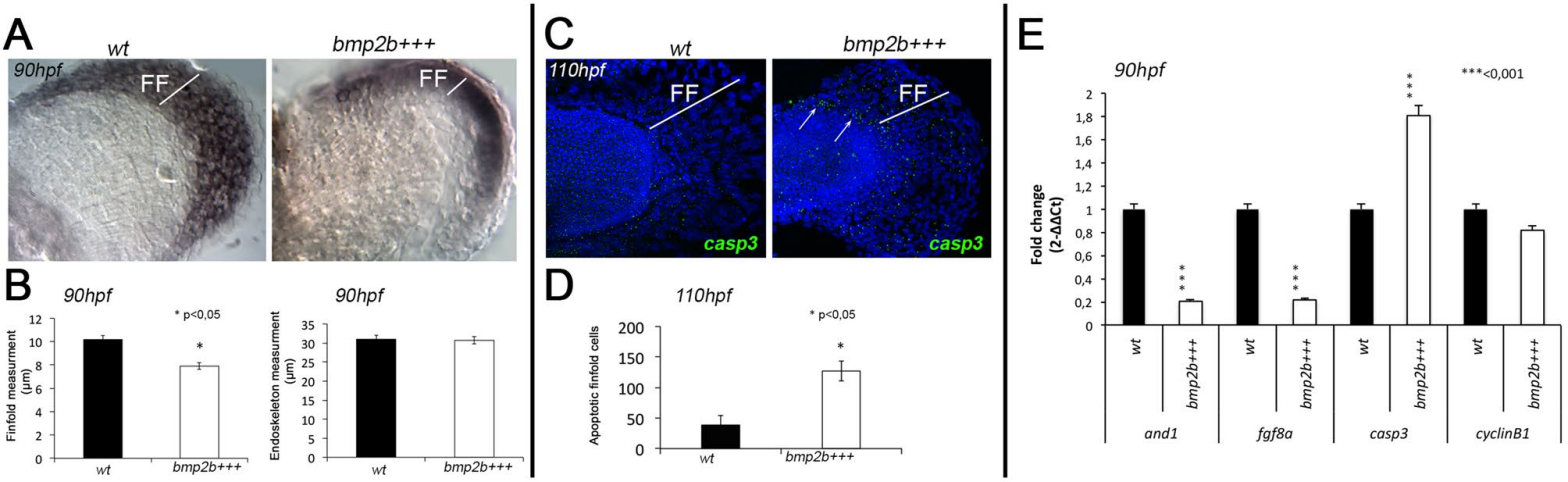
Phenotype and gene expression during fin development in *bmp2b*-overexpressing fins (*bmp2b*+++) and controls (*Wt*). (**A)** ISH for *and1* highlights the shorter finfolds (FF) of *bmp2b*+++ fins in comparison with controls, similar to *hoxd13*+++ *fins*. (**B)** Finfold and endoskeleton measurements, after *and1* ISH, revel significant reduction of the finfold in *bmp2b*+++ *fins* (n = 50; *p < 0.05). (**C,D)** Imunnostaining with anti-casp3 antibody **(C)** and subsequent counting of the casp3-positive cells in the finfold (n = 9; *p < 0.05) **(D)** suggests higher apoptotic activity in *bmp2b*+++ finfolds than in *Wt* controls at stage 110 hpf. (**E)** RT-qPCR analyses to evaluate gene expression in *bmp2b*+++ fins and *Wt* controls at 90 hpf indicate upregulation of *casp3* (***p < 0.001), downregulation of finfold markers *and1* and *fgf8a* (***p < 0.01) and non-significant alteration of *ccnb1*, involved in cell proliferation. Statistical significance evaluated by unpaired t-test.

Taking into account the upregulation of several *bmps* in *hoxd13a*-overexpressing fins, we hypothesized that increased levels of *hoxd13a* may trigger *bmps* expression influencing finfold development. To gain insight into this hypothesis, we selected *bmp2b* for further functional testing, given that the tetrapod orthologous is involved in the formation of the AER^44^. We generated a transgenic zebrafish line, in which *bmp2b* was placed under the control of an *hsp70* promoter and induced overexpression through heat-shock treatments at 32hpf, following the proceedings used for the transgenics allowing *hoxd13a* overexpression. We were able to identify finfold reduction after *bmp2b* overexpression not only in the pectoral fins but also in the caudal fin in 59,3% of the embryos (Supplementary Information: Fig. 3). We next performed ISH using the finfold marker *and1* and measured both the finfold (stained region) and the fin endochondral plate (Fig. 3A). We found a significant reduction of the finfold in *bmp2b*-overexpressing fins (n = 50), while no significant differences were found in the size of the endoskeletal plate (Fig. 3B).

We next, explored potential mechanisms involved in the finfold reduction observed in *bmp2b*-overexpressing fins. To evaluate if finfold reduction was due to increased cell death, we immunostained zebrafish embryos with anti-casp3 antibody and detected that *bmp2b*-overexpressing fins present a higher number of casp3-positive cells than controls (n = 9; Fig. 3C,D), suggesting a higher rate of apoptosis. In addition, we also evaluated the expression of *casp3* by RT-qPCR in the developing fins*,* together with fgf8, and1, and ccnb1 (Fig. 3E). We found that, while the expression of finfold markers diminished significantly in *bmp2b-*overexpressing fins (*fgf8a* and *and1*), the expression of *casp3* significantly increased and the expression of *ccnb1* did not change significantly at 90hpf. These data suggest that *bmp2b* overexpression reduces the signaling activity of the finfold, which is required for the maintenance of an undifferentiated state in the underlying mesenchyme in tetrapod models^45^. It also reduces the expression of a gene (*and1*), which encodes the actinotrichia proteins required for finfold development^13^. Moreover, the higher expression of *casp3* in *bmp2b-*overexpressing fins suggests increased cell death in the finfold, which may justify its reduced size in the transgenic condition. Interestingly, reduced expression of *bmp2* was reported to be associated with dorsal and ventral expansions of the AER in the mutant mice (Megf7-deficient), which also presents reduced apoptotic activity^46^. Thus, while the overexpression of a *bmp2* orthologous in zebrafish cause reduction of the finfold, its reduced expression in mice causes AER expansion and both processes relate with an impact on apoptosis.

### Long-fin leo^t1^/lof^dt1^ mutants express less *hoxd13a* and *bmp2b* during fin development

**Figure 4:**
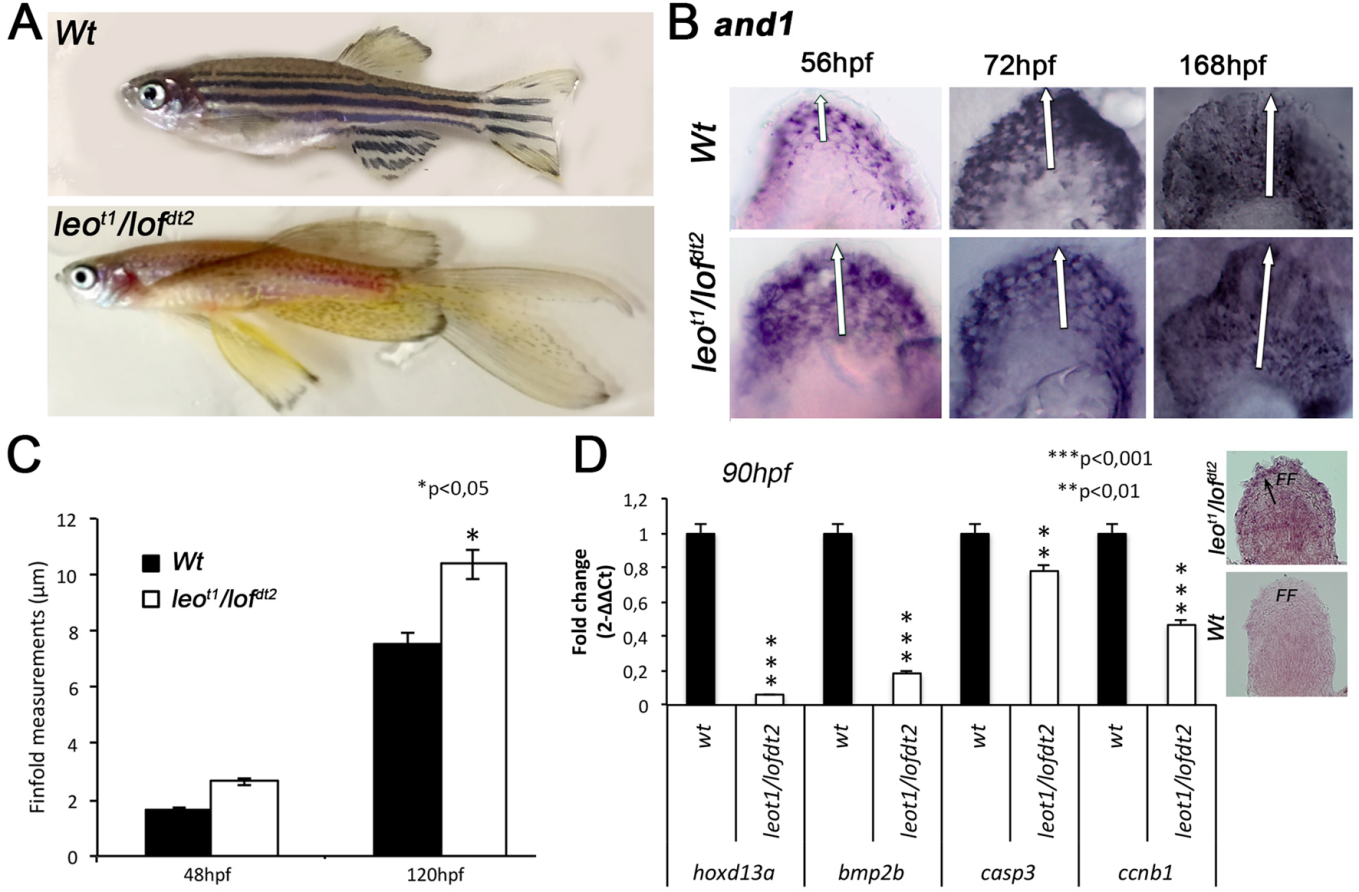
Finfold growth and gene expression in leo^t1^/lof^dt2^ mutants and controls (*Wt*). **(A)** Fin phenotype comparisons in adults. (**B,C)** Finfold size comparison during development evaluated after *and1* ISH **(B)**, followed by measurements **(C)**, revealing a longer finfold detectable since 48hpf in leo^t1^/lof^dt2^ mutants, in comparison with controls (n = 20, *p < 0.05). (**D)** Gene expression analyses by RT-qPCR at 86hpf, suggest lower expression levels for *hoxd13a* and *bmp2b* in leo^t1^/lof^dt2^ fins in comparison with *Wt* controls (***p < 0.001) detected by RT-qPCR (on the right) and ISH (on the left), which was accompanied by decreased expression of *casp3*, involved in apoptosis (**p < 0.01) and *ccnb1,* involved in proliferation (***p < 0.001). Statistical significance evaluated by unpaired t-test.

The zebrafish mutant leo^t1^/lof^dt1^ exhibits a phenotype characterized by long finfolds (Fig. 4A). To further investigate the relationship between *hoxd13a/bmp2b* and finfold size, we analyzed finfold development in this mutant in comparison with WT controls (Fig. 4B–D). We performed ISH for the finfold marker *and1* and measured the stained regions (Fig. 4B). We found that leo^t1^/lof^dt1^ mutants show longer finfolds detectable from 48hpf onwards (n = 20, Fig. 4B,C). In addition, the expression of *hox13a* and *bmp2b* was lower in these appendages than in the wild-type condition at 86hpf, which was accompanied by lower expression of *casp3* and *ccnb1* (Fig. 4D). Thus, while the shortening of the finfold in our transgenic conditions seems to be associated with increased levels of *hoxd13a* and *bmp2,* the long finfold of leo^t1^/lof^dt1^ mutants seem to be associated with lower levels of *hoxd13a* and *bmp2b.* This is consistent with our hypothesis suggesting that increased levels of *Bmp2*, mediated by the Hoxd13 transcription factor, may have been an important mechanism involved in the shortening of the finfold during evolution.

## Discussion

Modulation of *Hoxd13*, through the elaboration of its enhancer network, has been suggested as one of the mechanisms involved in the transition from fish fins to tetrapod limbs^32^. Taking advantage of the current knowledge on Hoxd13 targets in tetrapods^34^, we tested how the overexpression of the zebrafish orthologous (*hoxd13a*) throughout zebrafish fin development impacts the expression of a set of 10 putative downstream targets. We found that most of these genes presented altered expression levels when in *hoxd13a-*overexpressing fins. The overexpression of *hoxd13a* leads to the downregulation of genes involved in the establishment of the proximal identity, such as *meis1* and *emx2*, Thus, in addition, to instigating the distal cells for the fins to enter a distal-identity program, as suggested by  Freitas and colleagues^32^, the overexpression of *hoxd13a* may also cause active repression of the proximal identity. Furthermore, previously chicken functional studies indicate that Dach1 participates in the repression of *Meis* genes, contributing to establish the identity of the distal limb domains^40^. Our ISH results for *dacha* (zebrafish *Dach1* orthologous), shows that transgenic zebrafish maintain *dacha* expression in the most distal area of the fins, which suggests that downregulation of *meis1* in *hoxd13a*-overexpresing fins may result from direct or indirect regulation of *meis1* by hoxd13a transcription factor. In addition, in chicken, Dach1 is known as an antagonist of Bmp-mediated transcriptional control, and the cross-talk between those 2 genes is required for the PD patterning and maintenance of the AER^40^. Therefore, upregulation of *dacha i*n *hoxd13a*-overexpressing fins may contribute to the maintenance of an AER-like structure rather than allowing the expansion of the finfold, via interference with the bmp-signaling.

We also found that *fbn1* is up-regulated *hoxd13a*-overexpressing fins being ectopically expressed in the remaining finfold region. A similar distal increase of *fbn1* expression, in response to *Hoxd13* overexpression was also reported in chicken limb buds^34^. Given that *Fbn1* encodes a major component of extracellular microfibrils^42^, our data suggest that the distal endochondral plate extension observed in the *hoxd13a*-overexpressing fins^32^ may stem from a *hoxd13a* dependent control of *fbn1* expression.

*Barx1* is another transcription factor shown to be regulated by Hoxd13 transcription factor during chicken limb development and its missexpression causes *Barx1* downregulation in the autopod^34^. Our data show the inverse trend in *hoxd13a*-overexpressing fins: increase levels of *hoxd13a* lead to increased levels of *barx1*. Regarding *bmp* genes (*bmp2a, bmp2b*, *bmp4, bmp7a,* and *bmp7b*), we found significant upregulation of *bmp2b* and *bmp7b* at 85hpf*.* In addition, these genes appeared ectopically expressed in the remaining finfold of *hoxd13a*-overexpressing fins. Interestingly, *Bmp2* seems to limit the elongation of the AER in tetrapod models^41,47^ and its inhibition increases its size^48^. This led us to hypothesize that the finfold phenotypes observed in *hoxd13a*-overexpressing fins result from alterations in BMPs activity and, in fact, when we overexpressed *bmp2b* we were able to obtain equally finfold truncation. In addition, complementary assays suggested that *bmp2b* overexpression generated this phenotype by incrementing apoptosis and downregulating fgf8 signaling*,* crucial for the survival of AER cells in tetrapod models^49^. Additional support for our hypothesis, suggesting involvement of hoxd13a/*bmp2b* interactions in finfold size definition, was obtained analyzing the formation of the long finfolds of leo^t1^/lof^dt1^ mutants, in which the expression of *hoxd13a* and *bmp2b* seems to be lower compared to controls, as well as the apoptosis in these structures.

Our data hint that Hoxd13 contribution to the fin-to-limb transition may have resulted from suppression of proximal identity determinants (*meis1*, *emx2, barx1*) and upregulation of genes required for PD limb patterning (*dacha*) and for distal skeletogenesis (*fbn1*). We also present evidence that supports the hypothesis that finfold loss may have been achieved during vertebrates’ evolution through modulation of Bmps distally mediated by Hoxd13, which may have increased the apoptotic levels inhibiting finfold elongation.

## Methods

### Zebrafish maintenance and manipulation

Zebrafish experiments followed European Union Animal Research Guidelines and the experimental design was approved by the Ethics Committee of IBMC/I3S and DGAV (Portugal). Embryo staging followed Kimmel et al.^50^ and transgenesis followed previously described methods^32,51^. Heat-shock treatments were performed in *hsp70:hoxd13a* and *hsp70:bmp2b* lines and in AB WT at 30 hpf. To this end, three sequential heat-shock treatments were conducted at 36, 48, and 60 hpf, by placing embryos at 38.5 °C for 60 min (min.). They were then fixed in 4% PFA for ISH and Immunohistochemistry or used to collect fins for RNA extraction, subsequent cDNA synthesis, and RT-qPCR analyses.

### Generation of the *hsp70:bmp2b* zebrafish transgenic lines

A construct harboring *bmp2b*, placed under the control of the heat-shock inducible *hps70* promoter, was used to generate zebrafish transgenic line *hsp70:bmp2b*, allowing *bmp2b* overexpression at 30hpf. The coding sequence of *bmp2b* was isolated by PCR using the primers *bmp2b_Fwd* 5′-cggaactgactgatcatggtc-3′; *bmp2b_Rev* 5′-ggagattgttctcatcggcac-3′ and cloned into the pCR8/GW/TOPO vector. Correctly orientated clones were used as middle entry vectors to generate Tol2kit constructs^52^ along with the promoter of hsp70 (5′ entry vector), PolyA (3′ vector), and pDESTtol2CG2 as the destination vector. This vector contains a cmlc2:egfp transgenesis marker that promotes GFP expression in the heart, facilitating the identification of transgenic embryos^52^.

### Whole-mount ISH and immunohistochemistry

Digoxigenin-labeled riboprobes were generated using a dig-UTP labeling mix and T3, T7, or SP6 RNA polymerases according to the manufacturer’s instructions (Roche). ISH was carried as previously described^32,53^. Immunofluorescence protocol was adapted from Mateus and colleagues^54^ with the following adjustments: embryos were fixed in 4% paraformaldehyde at 4 °C overnight (o/n) and then dehydrated in increasing levels of MeOH (methanol) in PBT (PBS and 0.1% Tween) and stored in 100% MeOH at – 20 °C. Embryos were then rehydrated in reverse series of MeOH /PBT solutions and washed twice in PBT for 5 min. at room temperature (RT), followed by permeabilization with 100% Acetone for 7 min. at – 20 °C. Next embryos were washed in PBT, 0.1% DMSO and 1% Bovine Serum Albumin (BSA) and subsequentially blocked in PBT, 1% DMSO, 1% BSA, and 1% goat serum for 2 h at RT. Embryos were incubated in primary antibody (rabbit anti-PSmad1/5/9, 1:100, Cell Signaling) diluted in blocking solution at 4 °C o/n. The following day embryos were washed 2 times in PBT, 0.1% DMSO, and 1% BSA for 5 min and incubated in secondary antibody (Alexa Fluor 568 anti-rabbit, 1:1000) conjugated with DAPI (1:1000) (Sigma) diluted in blocking solution o/n at 4 °C. On the next day embryos were washed 2 times in PBT, 0.1% DMSO, and 1% BSA for 5 min. Embryos were mounted in 80% Glycerol, 2% DABCO (Sigma) diluted in PBS, and then imaged in Leica SP5II confocal microscope.

### RT-qPCR

RNA was extracted from pools of dissected fins (n = 100 per experimental condition) and converted into cDNA using the High-Capacity RNA-to-cDNA Kit, Thermo Fisher Scientific). RT-qPCR was performed in triplicates for each analyzed gene using *Rpl13a* and *β-actin* as reference genes and primers indicated in Supplementary Information (Table 1). Data were analyzed using Bio-Rad iQ5 Optical System Software Version 2.0 software, and relative gene expression quantification was calculated using the 2-ΔΔCt method. Statistical significance was calculated by Unpaired T-test.

## Supplementary Information

Supplementary Information.
