# Supplementary material for: Hoxd13/Bmp2-mediated mechanism involved in zebrafish finfold design
Source: Sci Rep. 2021 Mar 30;11:7165. doi: 10.1038/s41598-021-86621-4 (PMC8009906; doi:10.1038/s41598-021-86621-4)
Supplement: Supplementary file 1 — Supplementary Information. [file 41598_2021_86621_MOESM1_ESM.pdf]

SUPPLEMENTARY INFORMATION

Title: Hoxd13/Bmp2-mediated mechanism involved in zebrafish finfold design

Author's list:

João Castro
Vanessa Beviano
Ana Paço
Joana Leitão-Castro
Francisco Cadete
Miguel Francisco
Renata Freitas

Supplementary Figure 1

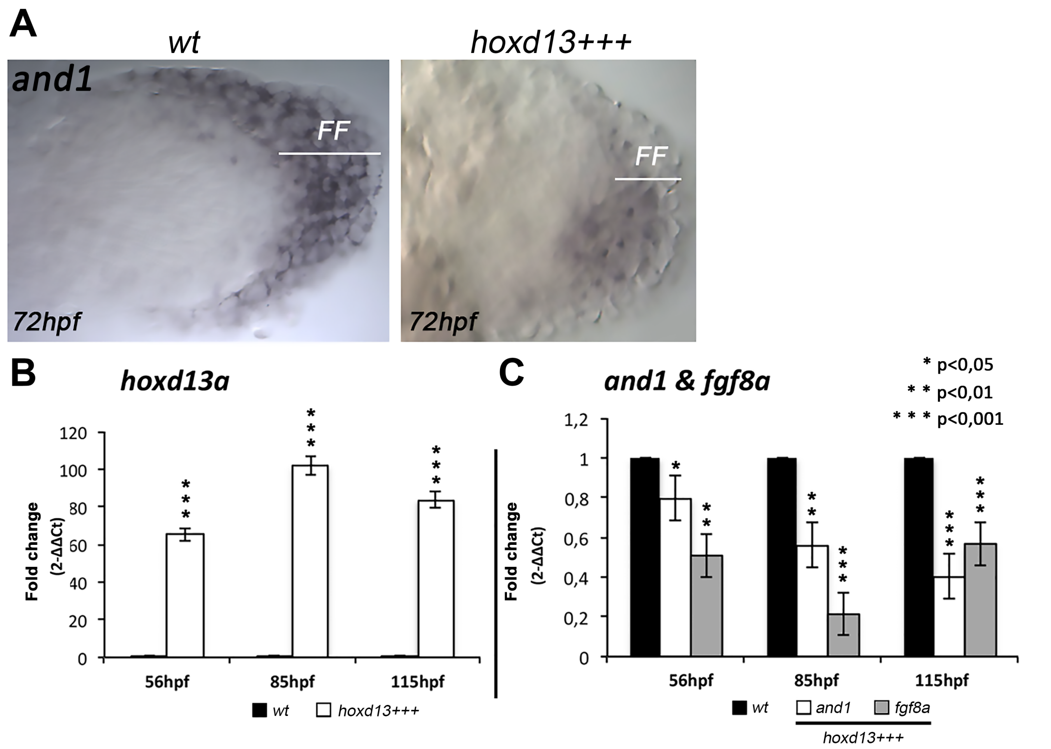

Supplementary Figure 1: Finfold growth and gene expression in wild-type (wt) and *hoxd13a*-overexpressing fins (*hoxd13+++*). A. ISH for the finfold (FF) marker *and1* highlights the reduction of this structure in the transgenic condition. **B.** RT-qPCR for *hoxd13a* reveals higher expression levels in the transgenic condition in three developmental stages. **C.** RT-qPCR for *and1* and *fgf8a* suggests a reduced expression in the transgenic fins in three developmental stages.

Supplementary Figure 2

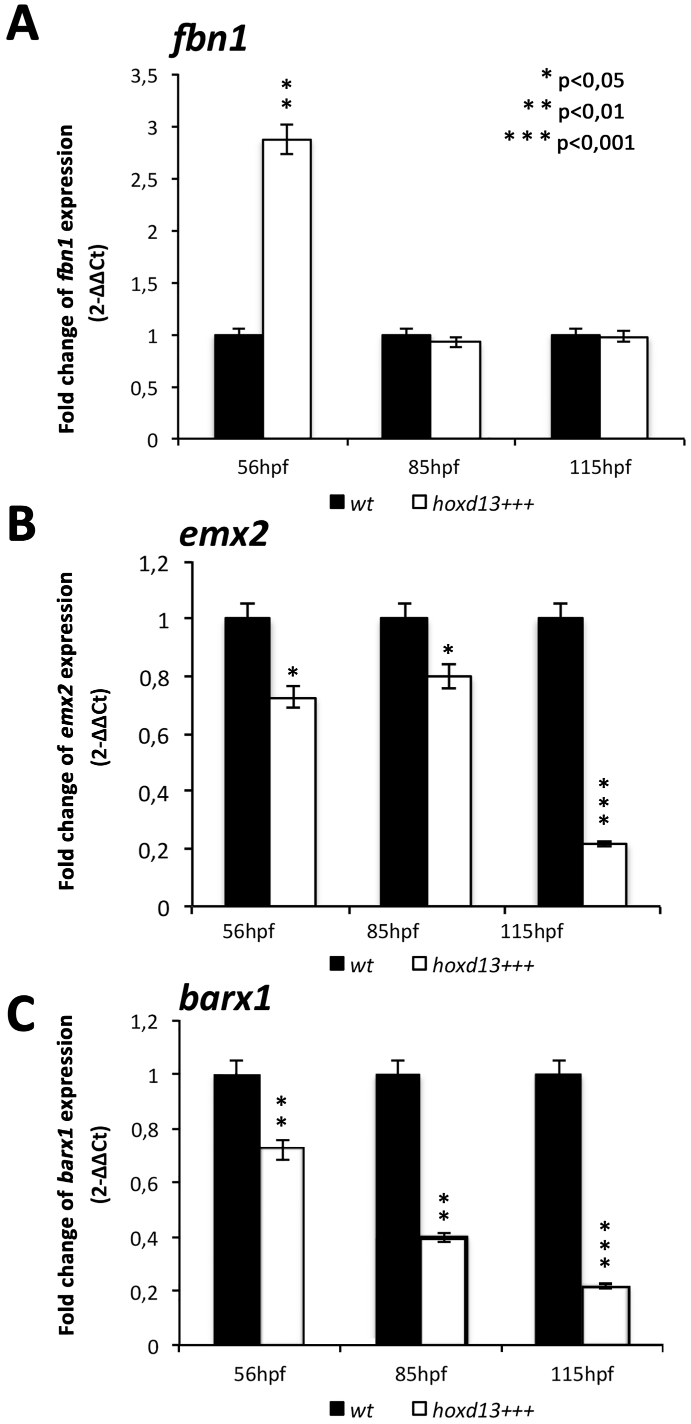

Supplementary Figure 2: Expression of putative *hoxd13a* targets in wild-type (wt) and *hoxd13a*-overexpressing fins (*hoxd13+++*) evaluated by RT-qPCR. A. *fbn1* appears to be upregulated in *hoxd13a*-overexpressing fins at stage 56hpf. **B-C.** *emx2* (B) and *barx1* (C) expression levels are significantly lower in *hoxd13a*-overexpressing fins, especially at 115hpf.

Supplementary Figure 3

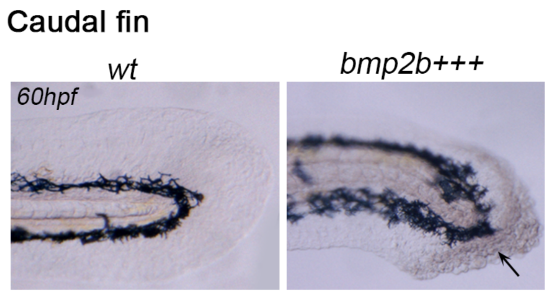

Supplementary Figure 3: Phenotype of the caudal finfold in *bmp2b*-overexpressing embryos (*bmp2b+++*) and controls (*wt*). Note reduction of the caudal finfold (arrow).

Supplementary Table 1

GENE		SEQUENCE 5´-3´
and1	F	CTTGAGGAAGCCCCAAAGAAACTG
	R	CATCTTGTAGGAGCGACGTGACT
β-actin2	F	ACTGTATTGTCTGGTGGTA
	R	TACTCCTGCTTGCTAATCCACATC
barx1	F	CTGGCAGAGTCCTTAGGCTTGAGT
	R	TCACTGGTGGGGATGGAGTTCTTTT
bmp2a	F	GCCAGCAGAGCCAACACTATC
	R	GATGGAGGTCAGGTTGAAGAGGAA
bmp2b	F	CGCCCGAGGAGCACTATG
	R	CAGGAATGGAGGTAAGGTTGAAGAA
bmp4	F	CACCGTGAGAGGATTCCAT
	R	GTCTGAGAAGGCGTCATCTATT
bmp7a	F	CCACAGAGGGTGCCAGCATAGA
	R	GATCCAGTCCTGCCATCCCAGAT
bmp7b	F	GACCTTCCGCATCAGCATCTACC
	R	AAACCAGCCAGCCCTCTTCT
casp3	F	AGAACTGGATCCTGGTGTGG
	R	GAGCCGGTCATTGTGTTTCT
ccnb1	F	CAGGCTTTGAAGAAGAAGGAGG
	R	GGCTCAGACACAACCTTAACG
dacha	F	AGAGTCATCTCCTGGCTAAC
	R	GGTGGTTCATCTGGTTCATT
dachb	F	CACAAGACTCACAATGGCTCCGAAT
	R	TGAAGCAGGTGATGAATGTTTCTCTCT
dachc	F	CCAGAGTGTCCCGTCCAGAATG
	R	GACCTGCGACCGTCATCCAA
emx2	F	CGCACAGTCCGCACCCTCTT
	R	TCCGCACCGACGACGTAATG
fbn1	F	GGTCTGTCTTCATCTGGTCCTC
	R	GTGTTGCGTTGGCGTCTC
fgf8a	F	TTGCGTTTTGCTACTATGCTCAGGTA
	R	TTGCCACTGGTTCGGCTGTA
hoxd13a	F	CGGAGCCCGCATATCTGGAA
	R	CGATCCGCCGTCTGTTCTCC
meis1b	F	CAACCGAGCAGTAAGTCAAG
	R	ATTCCCATCCCACTCATAGG
rpl13a	F	CTCGGTCGTCTTTCCGCTATTGTG
	R	AAGATTCTGCTGGGGGCTCTGAAG

Supplementary Table 1: Sequence of the primers used in RT-qPCR reactions (Annealing Temperature: 62°C).

Supplementary Table 2

Genes	Stage (hpf)	Experimental conditions (ΔCT)	
		wt	***hoxd13+++***
meis1b	85	3,557811401	3,906994091
	115	4,421279468	5,34636664
dacha	56	7,380687339	6,420430505
	85	8,352151103	6,570549344
bmp2a	85	8,141533552	7,995935339
bmp2b	85	7,876302099	7,109933705
bmp4	85	6,823113758	6,672310971
bmp7a	85	10,64936778	10,98045523
bmp7b	85	7,744261898	6,380333894
fbn1	56	9,077422728	7,55509199
	85	6,197351276	6,311612786
	115	6,730047496	6,765635813
emx2	56	8,110033835	8,569115319
	85	7,900129376	8,225587295
	115	7,705342034	7,802735705
barx1	56	4,234286724	4,709252907
	85	4,983153874	6,314313468
	115	6,393333333	8,603333333
		wt	***bmp2b+++***
and1	90	0,656666667	2,95
fgf8	90	7,24	9,45
casp3	90	3,956666667	3,103333333
		wt	***leo^{t1}/lof^{dt2}***
hoxd13a	56	7,300044871	11,66693275
bmp2b	56	8,121841677	9,556963175

Supplementary Table 2: Gene expression non-normalized results obtained by RT-qPCR and corresponding to the **ΔCT** average of 3 biological replicates, each one obtained from a pool of dissected fins (n=100): wild-type fins (wt), *hoxd13a*-overexpressing fins (*hoxd13+++*), *bmp2b*-overexpressing fins (*bmp2b+++*) and mutant fins (leo^{t1}/lof^{dt2}).
